# Novel and Efficient Synthesis of Phenethyl Formate via Enzymatic Esterification of Formic Acid

**DOI:** 10.3390/biom10010070

**Published:** 2020-01-01

**Authors:** Minguk Shin, Jeongbae Seo, Yesol Baek, Taek Lee, Min Jang, Chulhwan Park

**Affiliations:** 1 Department of Chemical Engineering, Kwangwoon University, Seoul 01897, Korea; smg0324@naver.com (M.S.); bedan1203@gmail.com (J.S.); isss9511@naver.com (Y.B.); tlee@kw.ac.kr (T.L.); 2 Department of Environmental Engineering, Kwangwoon University, Seoul 01897, Korea; minjang@kw.ac.kr

**Keywords:** formic acid, phenethyl formate, esterification, lipase, enzyme reuse, value-added chemical

## Abstract

Current methods for the production of esters, including chemical synthesis and extraction from natural sources, are hindered by low yields and environmental pollution. The enzymatic synthesis of these compounds could help overcome these problems. In this study, phenethyl formate, a commercially valuable formate ester, was synthesized using commercial immobilized lipases. The effects of specific enzymes, enzyme concentration, formic acid:phenethyl alcohol molar ratio, temperature, and solvent were studied in order to optimize the synthesis conditions, which were identified as 15 g/L of Novozym 435 enzyme, a 1:5 formic acid:phenethyl alcohol molar ratio, a 40 °C reaction temperature, and 1,2-dichloroethane as the solvent. Under these conditions, phenethyl formate was obtained in a conversion yield of 95.92%. In addition, when 1,2-dichloroethane was replaced with toluene as the solvent, the enzyme could be recycled for at least 20 reactions with a steady conversion yield above 92%, testifying to the economic aspects of the process. The enzymatic synthesis of phenethyl formate using the proposed method is more environmentally friendly than methods currently employed in academic and laboratory settings. Moreover, the method has the potential to enhance the value-added properties of formic acid owing to its downstream use in the production of commercially essential esters.

## 1. Introduction

Carbon oxide (CO_x_) gases (CO and CO_2_) are key components of industrially produced syngas. These compounds directly and indirectly contribute to the effects of global warming, and a range of methods has been developed for eliminating or repurposing the gases into valuable chemical reagents. For example, microorganisms have been used to convert CO and CO_2_ in syngas into formic acid [1,2,3]. Although formic acid is vital for the production of a variety of materials across multiple industries, its low cost decreases the economic efficiency of these novel conversion processes, making it necessary to enhance the value-added properties of formic acid if these techniques are to be applied. In this context, the synthesis of formate esters from formic acid has been extensively studied [4,5]. Formate esters are notably more expensive than formic acid (approximately 50 to 100 times greater value addition) [6], and thus, various methods have been developed for their synthesis (Table 1) [4,5,7,8,9,10].

Esters, such as formate ester, are generally synthesized by the esterification of carboxylic acids with alcohols. Many esters are commercially valuable chemicals regularly employed in the cosmetic, resin, biofuel, plastic, and surfactant industries [11]. Esters commonly exhibit a characteristic fruit-like odor, and some, such as flavor esters, are used in shampoos and anti-aging creams, owing to their emollient, surfactant, and antioxidant properties [12]. One such formate ester, phenethyl formate, is a particularly widespread fragrance component found in cosmetics, perfumes, shampoos, and toiletries, with its usage in consumer products reaching 1–10 tons per year [13]. Esters, in general, play a valuable role in the fragrance and food additive industries, both of which are highly profitable and continue to experience growth globally [14].

It is essential that esters targeted for human consumption are nontoxic. However, the commercial production of esters generally employs strong acid catalysts, high temperatures (150–240 °C), and high pressures, which can result in compounds of low quality [15]. In addition, many of these procedures require extensive purifications, long reaction times, and specialized equipment to prevent corrosion during the reaction, all of which contribute to high production costs. Therefore, the enzymatic synthesis of esters under mild reaction conditions, as achieved with lipase, presents a promising alternative to these chemical processes [11,15].

Lipase is used in a variety of industries, including cosmetics and pharmaceuticals, and thus, its mechanism of action, reaction conditions, and structure has been extensively studied [16]. Lipases are superior to acid catalysts due to their wide range of substrate specificities. These enzymes are able to recognize substrate structures, be activated without cofactors, and function at room temperature and pressure [11]. However, as it can be difficult to separate lipases and reactants, the reaction conditions can hinder lipase activity. In addition, lipases are more expensive than their acid catalyst counterparts. Therefore, immobilized enzymes that are stable under various conditions are preferred for use in industry [17]. Recently, the synthesis of esters, such as isoamyl acetate, benzyl cinnamate, and phenethyl acetate, from organic acids and alcohols using immobilized enzymes has been reported [18,19,20].

**Figure 1:**
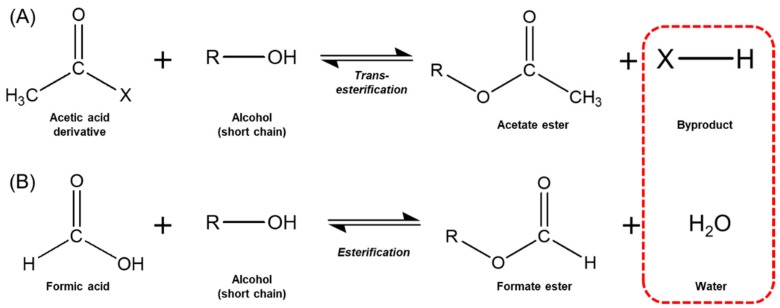
Byproducts (red dashed rectangle) from the transesterification of an acetic acid derivative (**A**) and the esterification of formic acid (**B**).

Although the esterification of a carboxylic acid with an alcohol primarily produces an ester and water, most processes use carboxylic acid derivatives or anhydrides to enhance conversion. Krishna et al. [18] reported that acetic acid binds to serine residues on the active sites of immobilized lipases, causing dead-end inhibition. In general, synthesis of acetic acid esters in high yields requires the use of acyl donors, such as acetate anhydride, ethyl acetate, or vinyl acetate. These acyl donors can be used for transesterification but unfortunately produce byproducts other than water (Figure 1) [20,21]. In particular, using vinyl acetate as an acyl donor decreases the yield of esters, owing to the formation of acetaldehyde, which can inactivate many lipases. One way to overcome the formation of these byproducts during transesterification is to use formic acid, as the only byproduct of direct esterification between formic acid and alcohol is water (Figure 1).

Achievement of smooth esterification and high yields of formate esters using standard methods requires specialized equipment, systems, and/or additives (Table 1). In addition, some of these methods, such as microwave irradiation, cannot be applied on a large scale; as such, developing high-yield, scalable methods for the enzymatic synthesis of formate esters using lipases could prove invaluable. Although lipase-based ester synthesis has been studied [11,15,19], little is known about the enzymatic synthesis of formate esters. For example, Janssen et al. [4] reported the lipase-catalyzed transesterification of ethyl formate with octanol to produce octyl formate. However, this transesterification required copious amounts of ethyl formate (ethyl formate:1-octanol molar ratio: 94:6) and excessively long reaction times to result in sufficient productivity (90% in 24 h).

In this study, we sought to address simultaneously two limitations of ester production, low productivity and toxic byproducts, by enzymatically synthesizing phenethyl formate. Phenethyl formate is an economically valuable formate ester, as it is harmless to humans (LD_50_ > 5000 mg/kg) and is a key ingredient used in the cosmetic and perfume industries, with an annual demand of ~1–10 metric tons [13,22]. Furthermore, phenethyl formate was recently cleared for use as a food additive for all animal species, as reports indicate that it is generally nontoxic [23]. To identify the optimal reaction conditions, enzyme type, enzyme concentration, molar ratio of substrates, temperature, and solvent were systematically studied. Enzyme reuse was also examined to investigate the economic aspects of the process. Overall, we determined that the synthesis of phenethyl formate using lipases is significantly more efficient and more eco-friendly than current methods. To the best of our knowledge, this is the first report on the synthesis of phenethyl formate using lipases.

## 2. Materials and Methods

### 2.1. Materials

Phenethyl alcohol (98%) and formic acid (99%) were purchased from Junsei (Tokyo, Japan) and Dae-Jung (Gyunggido, Korea), respectively. Novozym 435, Lipozyme RM IM, and Lipozyme TL IM were purchased from Novozymes (Bagsværd, Denmark), and Lipase PS Amano IM was purchased from Amano International Enzyme Co. (Nagoya, Japan). The following solvents were used in this study: acetonitrile (99.5%; Junsei), acetone (99.5%; Dae-Jung), tetrahydrofuran (THF, Dae-Jung), 1,2-dichloroethane (99%; Dae-Jung), toluene (99.5%; Junsei), cyclohexane (99.5%; Dae-Jung), *n*-hexane (96%; Junsei), heptane (98%; Dae-Jung), and isooctane (98%; Dae-Jung).

### 2.2. Standard Reaction Conditions

Theoretically, the esterification of 1 mol of formic acid with 1 mol of phenethyl alcohol produces 1 mol of phenethyl formate and 1 mol of water. It is therefore a 1:1 stoichiometric reaction. As such, formic acid (100 mM) and phenethyl alcohol (100 mM) were dissolved in a given solvent, and 10 mL of each solution was transferred to a 50 mL serum bottle. The total reaction volume was 20 mL with a final substrate concentration of 50 mM. Prior to the reaction, an immobilized lipase was added to the solution. As the volatilities of the solvent, the substrates, and the products can influence the reaction, the serum bottle was sealed by crimping. The reaction was then mixed for 4 h at 150 rpm using a shaking incubator (JEIO TECH.CO., LTD, Daejeon, Korea).

### 2.3. Reaction Condition Optimization

To identify the optimal conditions for phenethyl formate synthesis, the reaction parameters were analyzed in the following order: enzyme type, enzyme concentration, molar ratio of reactants, reaction temperature, and solvent selection. 

Four different immobilized enzymes (i.e., Novozym 435, Lipozyme RM IM, Lipozyme TL IM, and Lipase PS Amano IM) were used for the optimization [17]. Solutions of all the four enzymes with the same concentration and mass (10 g/L and 200 mg, respectively) were added to the serum bottles containing a 1:1 formic acid:phenethyl alcohol molar ratio. The solvent employed for these experiments was *n*-hexane, and the temperature was maintained at 30 °C.

To analyze the effect of the enzyme concentration on the reaction, the concentration of Novozym 435 was varied in a range of 5 to 30 g/L at an interval of 5 g/L. Reactions were performed using a 1:1 formic acid:phenethyl alcohol molar ratio (50 mM) at 30 °C with *n*-hexane as the solvent.

The molar ratio of formic acid to phenethyl alcohol was varied (1:1, 1:3, 1:5, 1:7, 1:9, and 1:11), and its effect on the product conversion was evaluated. Reactions were performed using Novozym 435 (15 g/L) at 30 °C with *n*-hexane as the solvent. 

Reactant conversion was analyzed at reaction temperatures of 20, 30, 40, and 50 °C. Reactions were performed using Novozym 435 (15 g/L) as the immobilized enzyme, a 1:5 formic acid:phenethyl alcohol molar ratio, and *n*-hexane as the solvent. Prior to the reaction, the reaction mixture without enzyme was preheated to the desired reaction temperature without agitation in a shaking incubator. 

The effect of the solvent on the reaction was studied. The optimal solvent was selected based on the log P value [18,24]. Acetonitrile (log P = −0.33), acetone (−0.042), THF (0.49), 1,2-dichloroethane (1.48), toluene (2.5), cyclohexane (3.4), *n*-hexane (4.0), heptane (4.27), and isooctane (4.6) were tested as solvents. Reactions were performed using Novozym 435 (15 g/L) as the immobilized enzyme and a 1:5 formic acid:phenethyl alcohol molar ratio at 40 °C.

### 2.4. Enzyme Reuse

To analyze the economic feasibility of the enzyme-catalyzed esterification reaction described in this work, enzyme reuse experiments were conducted after optimizing the parameters for the phenethyl formate synthesis. Enzyme reuse was investigated at the optimized conditions of a 15 g/L Novozym 435 concentration, a 1:5 formic acid:phenethyl alcohol molar ratio, a reaction temperature of 40 °C, and 1,2-dichloroethane and toluene as the solvents. Following the reaction, the enzyme was recovered by filtration and washed with *n*-hexane. The enzyme was then dried in a vacuum desiccator containing silica gel for 30–60 min and reused. A total of 20 cycles were conducted for each solvent.

### 2.5. Analytical Method

After 4 h, the samples (0.5 to 1 mL) were removed from the reaction mixture using a syringe. The samples were filtered into 2 mL vials using a filter-equipped syringe (PTFE, 0.2 µm, Advantec Co., Tokyo, Japan) to obstruct enzyme particles. Vial contents were analyzed on an Agilent 7890A gas chromatograph (GC; Agilent Technologies, Wilmington, DE, USA) equipped with a flame ionization detector (FID) and an INNOWax column (length 30 m, inner diameter 0.25 mm, film thickness 0.25 µm). The samples (1 µL) were injected in the column using nitrogen as the carrier gas. The temperature program of an oven was as follows: 80 °C for 1 min, 80–230 °C at a heating rate of 10 °C/min, 230 °C for 3 min, 230–250 °C for 1 min, and 250 °C for 1 min. The phenethyl formate peak was identified using a standard solution and eluted at 9.34 min. As the esterification is a 1:1 stoichiometric reaction, the conversion of formic acid to phenethyl formate was calculated in terms of the initial molar ratio of formic acid to phenethyl alcohol, shown as following:

(1) $$\mathrm{Conversion}\;\mathrm{of}\;\mathrm{phenethyl}\;\mathrm{formate}=\frac{\mathrm{mol}\;\mathrm{of}\;\mathrm{phenethyl}\;\mathrm{formate}}{\mathrm{mol}\;\mathrm{of}\;\mathrm{formic}\;\mathrm{acid}}\times 100\;\left(\%\right)$$

## 3. Results and Discussion

### 3.1. Enzyme Selection

Phenethyl formate was synthesized from formic acid and phenethyl alcohol using immobilized lipases. Immobilized lipases are preferred in these reactions, as free lipases tend to aggregate in solution owing to a high content of hydrophobic amino acids [25]. The activity of the immobilized enzyme is dependent upon a variety of conditions including substrates, solvents, and supports [26]. Generally, enzymes immobilized on hydrophobic supports have higher activity compared to free enzymes. However, immobilizing enzymes on hydrophilic supports may reduce the enzymatic activity and the solubility of hydrophobic substrates [27]. Four different commercial immobilized lipases on distinct supports were examined in this work: Novozym 435 (originated from *Candida antarctica* lipase B), Lipozyme RM IM (originated from *Rhizomucor mehei*), Lipozyme TL IM (originated from *Thermomyces lanuginosus*), and Lipase PS Amano IM (originated from *Burkholderia cepacia*) (Table 2) [28,29,30,31,32]. The respective conversion yields of formic acid to phenethyl formate using these lipases were 47.83% (Novozym 435), 0.28% (Lipozyme RM IM), 0.34% (Lipozyme TL IM), and 0.60% (Lipase PS Amano IM) (Table 2). From these results, Novozym 435 was determined to be the optimal lipase.

With the exception of Novozym 435, none of the tested lipases resulted in the conversion of the reagents. This may arise from the substrate specificity of these enzymes. Lipozyme RM IM and Lipozyme TL IM are well-suited for the esterification/interesterifcation of macromolecular fatty acids. For example, Abdulmaleka et al. [33] reported high conversions (77% and ~65%) of oleic acid to galactose oleate using Lipozyme RM IM and Lipozyme TL IM, respectively. These enzymes have also been used to synthesize biodiesel (fatty acid alkyl ester) from triglycerides via transesterification with alcohols, such as methanol [34]. However, when reacting with formic acid, the simplest and lowest-molecular-weight carboxylic acid, they showed no activity. In addition, these enzymes display 1,3-specific lipase. Lipozyme RM IM and Lipozyme TL IM were reported to catalyze the esterification of carboxylic acids with the 1,3-hydroxyl groups of trihydric alcohols [29,33]. It is therefore possible that the 1,3-specific Lipozyme RM IM and Lipozyme TL IM did not convert phenethyl alcohol and formic acid to phenethyl formate due to a lack of regioselectivity in the reaction. 

The regioselective and stereoselective activity of the lipases could also explain the lack of activity from Lipase PS Amano IM. This enzyme has been reported to catalyze stereoselective acylation [35]. For this process, Lipase PS Amano IM would require an acyl donor, such as ethyl acetate or vinyl acetate, to transform the hydroxyl group of phenethyl alcohol to an acyl group. However, as neither formic acid nor phenethyl alcohol has stereocenters, these substrates may simply not be suitable reagents for Lipase PS Amano IM. 

The superiority of Novozym 435 over other immobilized lipases has been displayed previously. For example, Yadav and Devendran [11] reported that Novozym 435 displayed exceptionally high activity (conversion yield: 96%; reaction time: 1 h) in the synthesis of cinnamyl acetate from cinnamyl alcohol and vinyl acetate. In contrast, Lipozyme RM IM and Lipozyme TL IM resulted in conversion yields of 6% and 4%, respectively. The higher reactivity of Novozym 435 may result from its lack of specificity. In comparison with other lipases, Novozym 435 does not exhibit regiospecificity or stereoselectivity and can react with a wide range of substrates, including carboxylic acids as well as primary and secondary alcohols [36]. Owing to the absence of these features, Novozym 435 can synthesize phenethyl formate and reacts well with short-chain molecules [20].

### 3.2. Enzyme Concentration

**Figure 2:**
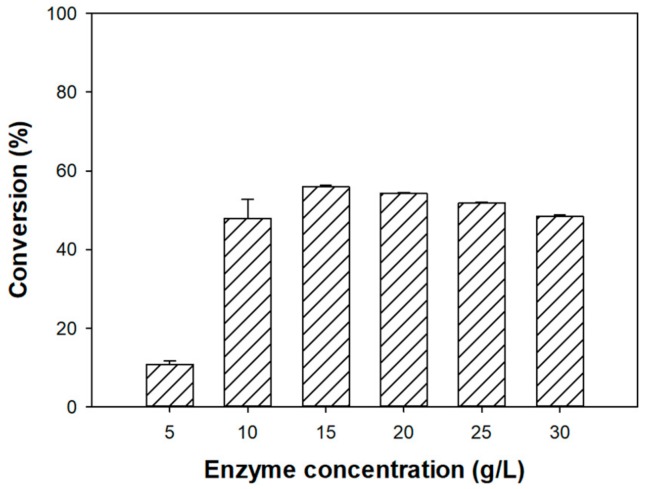
Effect of Novozym 435 concentration on the formation of phenethyl formate. The conversion yield (%) was calculated by GC analysis.

After identifying Novozym 435 as the optimal enzyme for the production of phenethyl formate, we sought to identify the optimal concentration for its esterification activity. At enzyme concentrations of 5, 10, 15, 20, 25, and 30 g/L, the conversion yields of the reagents to phenethyl formate were 10.68%, 47.83%, 55.87%, 54.17%, 51.78%, and 48.43%, respectively (Figure 2). The conversion initially increased with the increasing enzyme concentration, reaching a maximum at 15 g/L Novozym (55.87%); however, beyond this concentration, the conversion began to decline (Figure 2). 

These results can be understood in light of the properties of the immobilized enzyme. Novozym 435 is composed of *Candida antarctica* lipase B (CALB) adsorbed onto the surface of an acrylic resin carrier [37]. Phenethyl formate was synthesized at the active sites of Novozym 435. As the Novozym 435 concentration increased, the contact between the active sites and the substrates increased, and in turn, the conversion of phenethyl formate increased. However, due to the immobilization of the enzyme, a limitation of internal and external mass transfer could be expected at high enzyme concentrations, leading to a decrease in reaction efficiency [19]. For example, in the synthesis of benzyl cinnamate using Novozym 435 and Lipozyme TL IM, Wang et al. [19] reported that increasing the quantity of Lipozyme TL IM from 10 to 30 mg led to a rapid increase in the conversion of the substrates. However, with the quantity of enzyme above 30 mg, both the conversion and reaction rates decreased. Similarly, Sun et al. [28] observed an initial increase in conversion yield for the synthesis of erythorbyl palmitate using a Novozym 435 load from 1% to 15% (*w*/*w*), with a subsequent decrease in conversion when this loading was exceeded. This phenomenon is likely responsible for the observed decrease in the conversion of the reactants to phenethyl formate beyond the enzyme concentration of 15 g/L in our work.

Additionally, as described earlier, water is produced during the esterification of formic acid. This byproduct could adversely affect the conversion process by hydrolyzing the newly produced ester. In the presence of excessive enzyme, where substrates, products, and byproducts are in close proximity, this reverse reaction has an increased probability of occurrence and may have a noninsignificant contribution to the decreased conversion observed in this work [38,39].

### 3.3. Molar Ratio of Formic Acid to Phenethyl Alcohol

**Figure 3:**
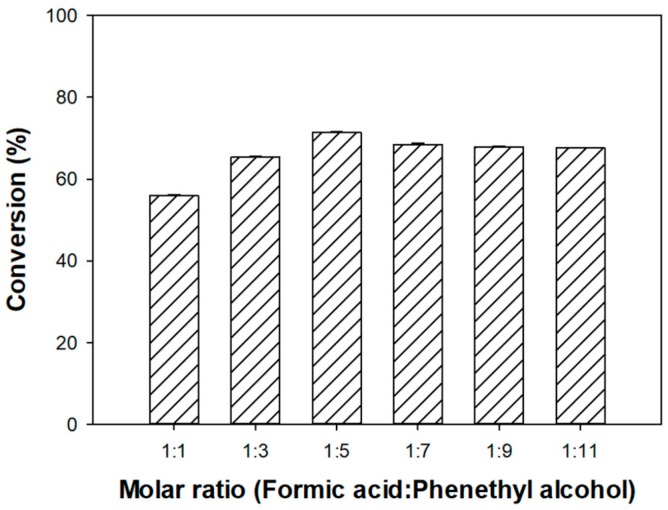
Effect of formic acid:phenethyl alcohol molar ratio on the Novozym 435-catalyzed synthesis of phenethyl formate. The conversion yield (%) was calculated by GC analysis.

We next investigated the effects of the formic acid:phenethyl alcohol molar ratio on the synthesis of phenethyl formate. For this study, the concentration of formic acid was set to 50 mM, and the phenethyl alcohol concentration was varied at an interval of 100 mM within a range of 50 to 550 mM. The formic acid:phenethyl alcohol molar ratios of 1:1, 1:3, 1:5, 1:7, 1:9, and 1:11 resulted in conversion to phenethyl formate with yields of 55.87%, 65.31%, 71.40%, 68.36%, 67.85%, and 67.62%, respectively (Figure 3). Conversion to phenethyl formate increased with an increasing molar ratio of phenethyl alcohol, reaching a maximum (71.40%) with a 1:5 formic acid:phenethyl alcohol molar ratio at the optimum point. Beyond this optimum point, the conversion values slightly decreased owing to the ping-pong bi-bi esterification mechanism of the lipase [40,41].

**Figure 4:**
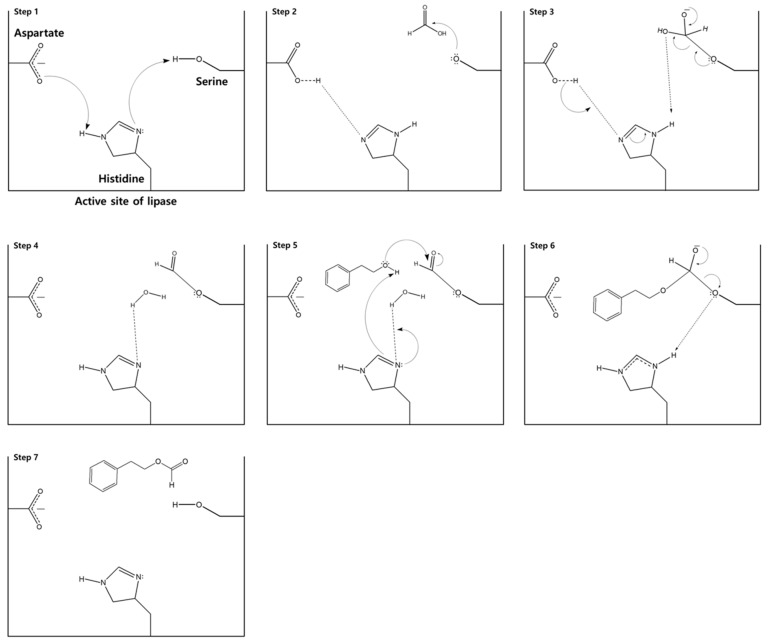
Mechanism for the lipase-catalyzed synthesis of phenethyl formate.

Lipase-catalyzed esterification begins with the formation of a nucleophilic serine in the active site of the lipase (Figure 4). Initially, the oxygen of an aspartate residue deprotonates histidine (step 1). The activated histidine then deprotonates the hydroxyl group of serine to form a nucleophilic oxide anion (step 2). The nucleophilic serine attacks the electrophilic carbonyl of formic acid to form a tetrahedral intermediate (step 3). The hydroxyl group of the intermediate deprotonates the histidine, forming water as a leaving group and an intermediate acylated enzyme. This is followed by an immediate capture of the leaving group water by the histidine (step 4). The nucleophilic oxygen of phenethyl alcohol subsequently attacks the acyl carbon of the enzyme intermediate, leading to the formation of a tetrahedral carbon substrate (step 5). This reaction occurs concomitantly with the histidine attack of the phenethyl hydroxyl hydrogen atom and the release of the histidine-bound water molecule. Electron reorganization in the tetrahedral intermediate leads to formation of the ester carbonyl group and attack of the histidine proton by the oxygen of serine (step 6). Finally, abstraction of the histidine proton releases the phenethyl formate product and returns the lipase to its original state (step 7).

Although the esterification of formic acid to phenethyl formate is essentially a bimolecular reaction, the conversion ratio differed with varying substrate molar ratios. This can be correlated to the reactions occurring at the active sites of the enzyme (Figure 4). Prior to the reaction with phenethyl alcohol, formic acid reacted at the active site of the lipase to form an intermediate acylated enzyme. Increasing concentrations of phenethyl alcohol meant that more substrates were present at the active site to react with the acylated intermediate, resulting in more product formation. However, the conversion was reduced, when the concentration of the alcohol exceeded the 1:5 optimum point, likely due to a phenomenon known as dead-end inhibition [30]. In enzymatic reactions, the ping-pong bi-bi mechanism results in the release of products following reaction with each of the individual reactants. Dead-end inhibition occurs during the ping-pong bi-bi mechanism, when the binding of one reactant at the active site of the enzyme inhibits the reaction between the second substrate and the enzyme [42]. It is possible that, beyond a certain phenethyl alcohol concentration, a phenethyl alcohol–enzyme complex is formed that prevents the ping-pong bi-bi mechanism from the proceeding stages, either by inhibiting the release of water from the formic acid–enzyme complex, or by directly preventing the reaction between the alcohol and the acyl intermediate (Figure 3). 

It should be noted that substrate-induced inhibition of commercial immobilized lipases has been reported. During the Novozym 435-catalyzed synthesis of ketoprofen ester using *n*-propanol, Duan et al. [43] observed a decrease in the reaction rate with increasing concentrations of *n*-propanol. This finding was directly linked to *n*-propanol-induced dead-end inhibition. Yadav and Trivedi [44] also identified dead-end inhibitions due to complexes formed between n-octanol and Novozym 435 during the synthesis of octyl acetate from n-octanol and vinyl acetate. In addition, Wang et al. [19] found that the lipase-catalyzed synthesis of benzyl cinnamate could be inhibited by high concentrations of benzyl alcohol. Beyond a 1:3 cinnamic acid:benzyl molar ratio, benzyl alcohol appeared to induce inhibition by distorting a water layer, which functioned in maintaining the lipase structure [19].

### 3.4. Reaction Temperature

**Figure 5:**
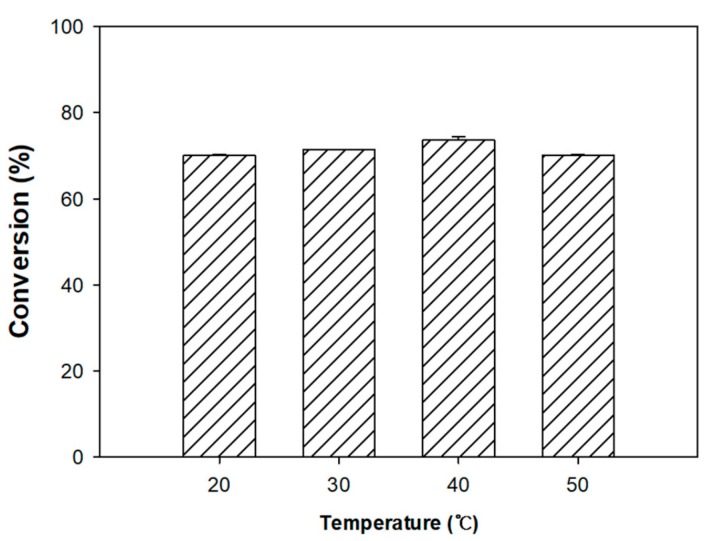
Effect of reaction temperature on the Novozym 435-catalyzed synthesis of phenethyl formate. The conversion yield (%) was calculated by GC analysis.

The effect of temperature on the lipase-catalyzed esterification of formic acid was also investigated. Esterification reactions were performed at 20, 30, 40, and 50 °C, resulting in conversion yields of phenethyl formate of 70.11%, 71.40%, 73.64%, and 70.05%, respectively (Figure 5). Although the highest conversion (73.64%) was obtained at 40 °C, only a slight difference (~3%) was observed between the highest and lowest conversion values. Therefore, the enzyme activity was not generally affected by the changes in temperature used in this work. This is in excellent agreement with the reported heat tolerance of Novozym 435 (20 to 110 °C) and studies, which have clearly shown good activity at 90 °C [28]. 

It is reported that the presence of a small volume of water is essential for lipase activity. A water layer around the lipase helps to maintain its three-dimensional (3D) structure, integrity, polarity, and stability [19,45]. Changes in temperature can affect the activity of water (a_W_), which can in turn affect the amount of water around the enzyme [24,45]. The initial esterification rate increased by increments of the kinetic constant due to the temperature raise, after which the rate of water production increased. According to theoretical moisture sorption isotherms, an increase in moisture content is accompanied by an increase in the water activity [46]. Therefore, the water layer around the lipase thickens owing to an increase in the amount of water. Although an appropriate water layer is important, a water layer with a great thickness could induce hydrolysis and thus inhibit substrate transfer. In contrast, an extremely low temperature can reduce the water activity, leading to an insufficient water layer for maintaining enzyme integrity [19]. Wang et al. [47] investigated conversion as a function of water activity during the synthesis of ethyl cinnamate. At a_W_ = 0.43, the highest conversion was obtained; low conversion values were observed at a_W_ ≈ 0 or a_W_ > 0.43. 

Based on those observations and the results from this work, the esterification reaction rate increased with an increase in temperature, but a thicker water layer caused hydrolysis and inhibited substrate transfer. In contrast, at low temperatures, when the reaction rate was decreased, the resulting lower water activity led to an insufficient water layer. Thus, the conversion of the reactants to phenethyl formate across the tested temperature range was essentially constant. However, as a reaction temperature of 40 °C yielded the highest conversion value, it was selected as the optimum reaction temperature (Figure 5).

### 3.5. Solvent Selection According to Log P

**Figure 6:**
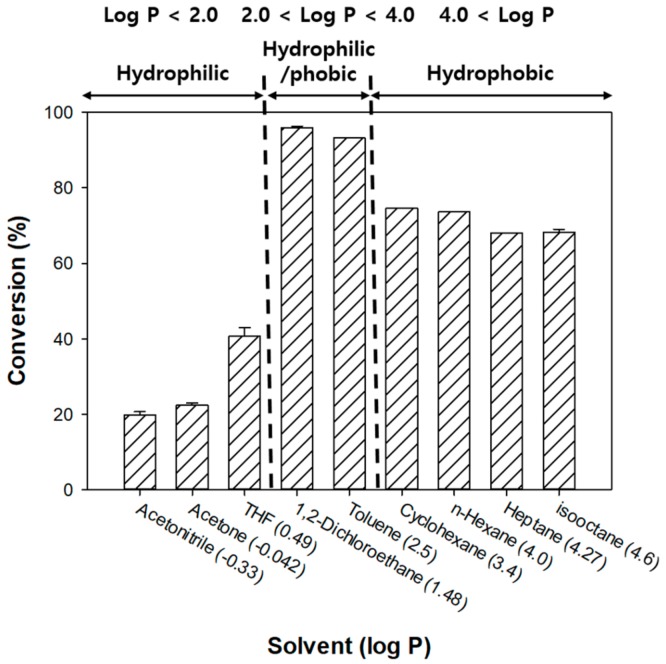
Effect of solvent log P on the Novozym 435-catalyzed synthesis of phenethyl formate. The conversion yield (%) was calculated by GC analysis.

Nine different solvents were chosen for the optimization of the Novozym 435-catalyzed esterification reaction. Almost all lipases have a hydrophobic lid attached to their active sites; i.e., they are activated, when the lid is opened in an organic solvent or oil–water surface, but not in an aqueous environment [48,49]. Therefore, only commonly used organic solvents were selected for the reaction [18,20]. In addition, the solvents were chosen to ensure that they would neither react with the substrates nor interfere with the activity of the enzyme. A comparison of the conversion yield with the log P value, which indicates solvent hydrophobicity, was used to determine the ideal solvent for the esterification reaction. The conversion values, in ascending order of log P values, were 19.79%, 22.43%, 40.64%, 95.92%, 93.18%, 74.59%, 73.64%, 68.04%, and 68.23% (Figure 6). The highest conversion (95.92%) was obtained with 1,2-dichloroethane, and with toluene, the second best conversion yield (93.18%) was achieved.

The solubility of the reactants and the products and the enzyme activity in a given solvent are the two primary routes, through which a solvent affects conversion. Therefore, it is important to diffuse substrates and products into the solvent [15]. Moreover, the effect of the solvent on the water layer and the protein structure, which in turn affects the enzyme activity, needs to be evaluated [50]. The solubility of the reactants and the products in a given solvent can be estimated in terms of log P. The log P values of formic acid, phenethyl alcohol, and phenethyl formate are −0.54, 1.36, and 1.876, respectively. The highest conversion yield was obtained with 1,2-dichloroethane, likely attributable to the similarity between its log P value and those of phenethyl alcohol and phenethyl formate, which had relatively high concentrations in the solution. Hydrophilic solvents can dissolve the water layer at the active sites of the enzyme and thus reduce its activity. Kamal et al. [51] studied the lipase activity in acetone and acetonitrile as representative polar organic solvents. They reported that an increase in the proportion of acetone and acetonitrile in the reaction mixture caused a reduction in the relative activity of the enzyme. Furthermore, organic solvents can affect the activity of water, leading to a distortion of the water layer involved in preserving the enzyme’s structure and activity.

The log P values of cyclohexane, *n*-hexane, heptane, and isooctane are 3.4, 4.0, 4.27, and 4.6, respectively, which indicate their strong hydrophobicity. Lipase activity can be maintained in these solvents, but water (as a byproduct) is accumulated and concentrated at the enzyme’s active sites, leading to ester hydrolysis and adversely affecting the conversion [10,18,42]. In addition, a highly hydrophobic solvent is not suitable for conversion owing to the insolubility of formic acid in these solvents [52]. Despite the negative effects on conversion of higher log P values, higher conversions were obtained with higher-log P-value solvents, likely due to the general stability of lipases in hydrophobic solvents [53].

From these results, it can be inferred that 1,2-dichloroethane and toluene have the appropriate log P values for Novozym 435-catalyzed esterification of formic acid. Using these solvents, the water layer of the enzyme could be maintained, and the substrate transfer was not limited by the diffusion of the reactants and the products.

### 3.6. Enzyme Reuse

**Figure 7:**
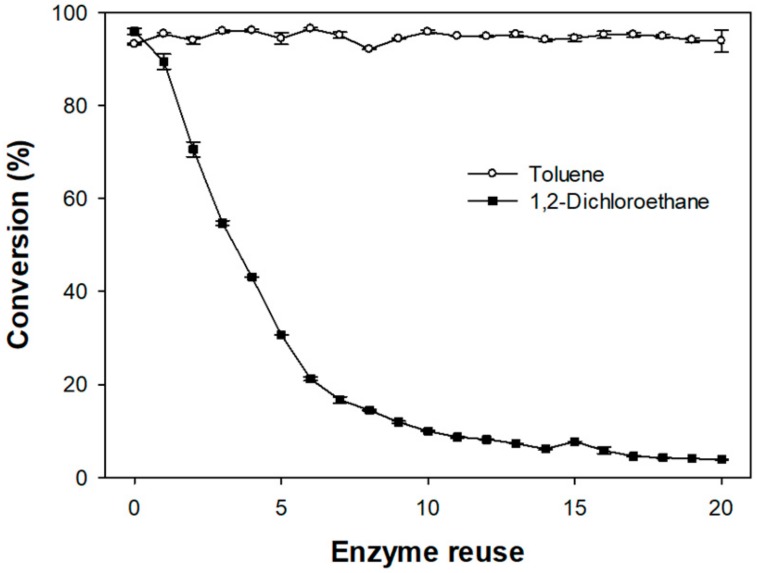
Recycling of Novozym 435 enzymatic activity in 1,2-dichloroethane and toluene. The conversion yield (%) was calculated by GC analysis.

In practice, enzymes are expensive, and thus enzymes that can be recycled multiple times are more beneficial for both laboratory and industrial applications. Manoel et al. [54] found that the activity of Novozym 435 was recyclable for esterification reactions, although after 192 h its activity gradually decreased. To determine whether this observation applied to our reaction system, we conducted enzyme reuse experiments under the optimized conditions of 15 g/L Novozym 435, a 1:5 formic acid:phenethyl alcohol molar ratio, a 40 °C reaction temperature, and 1,2-dichloroethane as the solvent. Unfortunately, the conversion yield reduced drastically, when the enzyme was reused in 1,2-dichloroethane and toluene, which was found to be the second best solvent for this reaction, was used. Toluene assisted in retaining the enzyme activity, which was able to be reused for 20 cycles, with a conversion level being maintained at a minimum value of 92% (Figure 7).

Even though 1,2-dichloroethane was the optimum solvent for the conversion, the enzyme reuse experiments showed the opposite result, owing to the instability of the lipase in the solvent. This may be due to the solubility of the immobilized enzyme support in the reaction solvent, which could lead to the enzymes becoming detached from the surface support. In addition, the solvent may distort the water layer of the lipase, or the enzyme could become inactivated due to the structural polarity of 1,2-dichloroethane [51]. Although a high conversion yield in 1,2-dichloroethane was observed for the initial reactions, it is assumed that the activity of the reused enzyme decreased as the water layer around the enzyme became distorted. Interestingly, when Valivety et al. [55] synthesized an ester from decanoic acid and dodecanol using a lipase, an exceptional reaction constant was observed with trichloroethylene, which has a log P value of 1.50 similar to that of 1,2-dichloroethane and also contains a chloride group. However, Liu et al. [56] demonstrated that, even when solvents have similar log P values, their functional groups could affect conversion during lipase-catalyzed ester synthesis. Thus, solvents affording high conversions might not be appropriate for maintaining lipase stability. Essentially, 1,2-dichloroethane was the appropriate solvent for achieving the highest conversion owing to its suitable log P value. However, the reused enzyme showed low conversion, likely arising from the 1,2-dichloroethane-induced distortion of the lipase.

In contrast, when toluene was used as the solvent, the activity of the enzyme was maintained over 20 cycles. This difference might be attributable to the different polarities of 1,2-dichloroethane and toluene. Using water as a reference, with its polarity set to 1, the relative polarities of 1,2-dichloroethane and toluene are 0.327 and 0.099, respectively. This low polarity of toluene, particularly with respect to 1,2-dichloroethane, can be attributed to its hydrocarbon nature and lack of halogen groups [57]. Therefore, in toluene, the water layer of the enzyme is not dissolved, and its activity can be maintained even with multiple reuses.

With respect to economic efficiency, it is important that esters can be purified using industrial processes. Lee et al. [58] studied the separation of acetic acid, water, and esters through azeotropic distillation. A commercial-scale process was designed, and they reported the separation of high-purity esters using *n*-heptane as an entrainer. Yamamura and Shimomura [59] studied the separation and purification of fatty ethyl esters using an industrial high-performance liquid chromatography (HPLC) system. They were able to obtain their compound with a 70% yield and a 99% separation purity using *n*-hexane as the solvent. Considering that toluene, like *n*-heptane and *n*-hexane, is a hydrocarbon, it is likely that esters synthesized in this solvent can also be separated with high purities.

## 4. Conclusions

Phenethyl formate was synthesized by direct esterification of formic acid with phenethyl alcohol using the commercial immobilized lipase Novozym 435. Following were the optimized conditions for the esterification of formic acid: a Novozym 435 concentration of 15 g/L, a 1:5 formic acid:phenethyl alcohol molar ratio, a 40 °C reaction temperature, and 1,2-dichloroethane as the solvent. Under these conditions, the conversion of formic acid to phenethyl formate was 95.92%. However, when 1,2-dichloroethane was employed in the enzyme reuse studies, enzyme denaturation occurred. Therefore, toluene, which showed the second highest conversion, was superior for the enzyme reuse analysis and provided a steady conversion yield of 92%, even after 20 cycles. The synthesis of phenethyl formate using enzymes is more efficient and more environmentally friendly than methods currently employed. Compared with existing synthetic methods such as transesterification, which can result in undesired byproduct formation, our esterification methodology only produces water as a byproduct. Owing to the high yield and the applicability of our reaction system, the production of vital formate esters from formic acid offers economic benefits to a variety of industries. In addition, these findings could help to promote the conversion of carbon oxides in syngas to formic acid, as this compound could then be exploited for the synthesis of novel and desired esters. 

## Figures and Tables

**Table 1:** Methods used for the synthesis of formate esters.

Alcohol	Product	Synthetic Method	Reaction Time	Reaction Conditions	Yield (Conversion)	Reference
*n*-hexanol;	Hexyl formate;	Microwave irradiation	160 s	PTSA in CH_2_Cl_2_	77%	[5]
Benzyl alcohol;	Benzyl formate;		220 s		85%	
Phenethyl alcohol	Phenethyl formate		180 s		81%	
Myristyl alcohol;	Myristyl formate;	Formylation	0.5 h	Reaction between thionyl chloride and DMF; Temp.: 0 °C	95%	[7]
Undecyl alcohol	Undecyl formate				96%	
Cyclohexanol	Cyclohexyl formate	Oxidation	1 h	Temperature (Temp.): 90 °C	45%	[8]
Methanol	Methyl formate	Methylotrophic yeast	90 h	NAD^+^-dependent dehydrogenation	90%	[9]
1-octanol;	Octyl formate;	Aerobic oxidation	10 h	Reaction between Catalytic Au/TiO_2_ and *p*-formaldehyde; Temp.: 80 °C	80%	[10]
Cyclohexanol;	Cyclohexyl formate;				90%	
Benzyl alcohol	Benzyl formate				55%	
1-octanol	Octyl formate	Enzymatic	24 h	Transesterification reaction with an ethyl formate:1-octanol molar ratio of 94:6	90%	[4]

**Table 2:** Properties of commercial immobilized lipases and values for the conversion^1^ of formic acid to phenethyl formate.

Immobilized Lipase	Specificity/Selectivity	Immobilization Support	Conversion ^1^
Novozym 435	Nonspecific	Acrylic resin (Lewatit vp oc 1600)	47.83%
Lipozyme RM IM	1,3-specific	Phenol–formaldehyde copolymer (Duolite ES 562)	0.28%
Lipozyme TL IM	1,3-specific	Silica gel	0.34%
Lipase PS Amano IM	Stereoselective acylation	Diatomaceous earth	0.60%

^1^ The conversion yield (%) was calculated by gas chromatograph (GC) analysis.
